# Supplementary material for: Characterization of a Marine Diatom Chitin Synthase Using a Combination of Meta-Omics, Genomics, and Heterologous Expression Approaches
Source: mSystems. 2023 Feb 15;8(2):e01131-22. doi: 10.1128/msystems.01131-22 (PMC10134812; doi:10.1128/msystems.01131-22)
Supplement: TABLE S1 [file msystems.01131-22-s0003.pdf]

Table S1 Expression levels of Pfam domians directly related with chitin in *Tara* Oceans Bacillariophyta metatranscriptomes.

Accession	PFAM transcriptioanl levels																															
	GO (Global Ocean)				IO (Indian Ocean)				MS (Mediterranean Sea)				NAO (North Atlantic Ocean)				NPO (North Pacific Ocean)				SAO (South Atlantic Ocean)				SO (Southern Ocean)				SPO (South Pacific Ocean)			
	0.8-5 μm	5-20 μm	20-180 μm	180-2000 μ	0.8-5 μm	5-20 μm	20-180 μm	180-2000 μ	0.8-5 μm	5-20 μm	20-180 μm	180-2000 μ	0.8-5 μm	5-20 μm	20-180 μm	180-2000 μ	0.8-5 μm	5-20 μm	20-180 μm	180-2000 μ	0.8-5 μm	5-20 μm	20-180 μm	180-2000 μ	0.8-5 μm	5-20 μm	20-180 μm	180-2000 μ	0.8-5 μm	5-20 μm	20-180 μm	180-2000 μm
PF01607	0.01496	0.062058	0.043722	0.0268	0.001392	0.005949	0.005347	0.012783	0.001263	0.003566	0.004814	0.000767	0.001719	0.010212	0.010468	0.005497	0.001271	0.00723	0.004833	0.000723	0.002679	0.019442	0.0081	0.004524	0.001237	0.000125	0.00017	0.000518	0.005399	0.015534	0.00999	0.001988358
PF03427	1.16E-05	5.97E-08	3.46E-08	0	0	0	0	0	0	0	0	0	0	0	0	0	0	0	0	0	9.34E-07	5.97E-08	0	0	1.07E-05	0	3.46E-08	0	0	0	0	0
PF02839	0.002393	0.000614	0.00016	0.000282	0.000286	1.41E-05	1.23E-05	3.85E-05	0.001335	0.000113	3.97E-06	1.48E-06	4.97E-05	7.34E-05	4.5E-06	1.14E-05	6.83E-06	2.71E-05	1.02E-05	0	0.000529	0.000145	5.47E-05	0.000222	6.93E-05	1.33E-05	2.37E-05	2.93E-07	0.000117	0.000227	5.03E-05	7.85473E-06
PF14600	0	0	9.41E-08	7.37E-07	0	0	0	0	0	0	0	0	0	0	0	0	0	0	0	0	0	0	0	0	0	0	9.41E-08	7.37E-07	0	0	0	0
PF00187	0.00292	0.004938	0.004626	0.096847	0.000186	0.000312	0.000871	0.026517	0.000334	0.000709	0.000934	0.00406	0.000381	0.000703	0.001288	0.007893	0.000194	0.000713	0.000466	0.035562	0.000529	0.000438	0.000502	0.002748	2.76E-05	9.46E-06	3.4E-05	1.89E-05	0.001269	0.002053	0.000532	0.020049287
PF00379	1.21E-05	0.000824	0.001427	0.01468	7.27E-07	0.000153	0.000224	0.002023	0	0.000237	0.000273	0.00318	5.3E-07	7.42E-05	0.000247	0.002214	6.68E-06	0.00028	0.000432	0.002098	0	1.34E-05	0.000106	0.000332	0	0	2.13E-06	0	4.18E-06	6.7E-05	0.000143	0.004833097
PF01644	0.000236	1.17E-06	6.33E-07	0	3.96E-08	0	0	0	1.74E-06	1.17E-06	5.02E-07	0	0	0	1.31E-07	0	0	0	0	0	0.000234	0	0	0	0	0	0	0	0	0	0	0
PF03142	0.002772	0.003426	0.00177	0.001652	0.000369	0.000197	0.000199	0.000136	0.000389	1.5E-05	6.81E-06	2.63E-05	0.000358	0.000623	0.000308	0.000324	0.000306	0.000272	0.000239	5.47E-05	0.000406	0.000885	0.000408	0.000495	1.75E-05	3.99E-05	5.17E-05	8E-05	0.000927	0.001394	0.000557	0.000536705
PF00704	0.014725	0.067178	0.138187	0.201512	0.003236	0.004853	0.017092	0.036766	0.002264	0.003471	0.032347	0.011679	0.001237	0.010602	0.034221	0.058658	0.002261	0.023461	0.011856	0.031295	0.0016	0.013962	0.033753	0.02952	0.000289	0.0001	0.000245	8.56E-05	0.003839	0.010729	0.008672	0.033508106
PF00182	0.001844	0.011732	0.025847	0.023812	0.00011	0.000984	0.004404	0.008218	6.62E-05	0.001168	0.006754	0.004459	0.000143	0.000538	0.001791	0.00534	0.000114	0.005548	0.006473	0.001919	0.000169	0.00092	0.001095	0.001221	5.66E-06	7E-07	8.88E-06	1.11E-06	0.001236	0.002573	0.005322	0.002653142
PF03067	0.054036	0.050734	0.0656	0.014521	0.005664	0.004079	0.006046	0.001387	0.003236	0.005619	0.006278	0.001761	0.008834	0.006259	0.013455	0.001056	0.00493	0.0099	0.008909	0.000416	0.012818	0.005837	0.010034	0.003467	0.001981	0.000204	0.000593	0.000393	0.016573	0.018836	0.020286	0.006041804
PF01522	0.00136	0.001289	0.000938	0.000223	0.000448	0.0002	5.84E-05	1.2E-05	9.99E-06	0.000268	0.000144	2.08E-06	1.84E-05	8.03E-05	0.000199	1.89E-06	0.000213	0.000141	2E-05	0	8.48E-05	0.000133	0.000151	4.49E-05	7.48E-05	2.39E-05	6.24E-05	3.11E-05	0.000511	0.000443	0.000304	0.000130529
