# Supplementary material for: Characterization of a Marine Diatom Chitin Synthase Using a Combination of Meta-Omics, Genomics, and Heterologous Expression Approaches
Source: mSystems. 2023 Feb 15;8(2):e01131-22. doi: 10.1128/msystems.01131-22 (PMC10134812; doi:10.1128/msystems.01131-22)
Supplement: TABLE S2 [file msystems.01131-22-s0004.pdf]

Table S2 All transcripts annotated as chitin synthase in *Thalassiosira pseudonana* genome from JGI database.

Transcript ID	No. of Intron	Full CDS	Full CDS correction	Transcript length (bp)	Chromosome location	Position	Correction
**4368**	0	Y		4374	4	chr_4:852844-857392 (-)	4368
22207	0	Y		4341	4	chr_4:852844-857392 (-)	i.e. 4368
27836	0	Y	N (incorrect ATG)	1572	4	chr_4:852986-854639 (-)	4368's partial sequence
27854	0	Y	N (incorrect ATG)	1932	4	chr_4:949547-951744 (-)	4413's partial sequence
22235	0	Y		3012	4	chr_4:949547-952816 (-)	i.e. 4413
**4413**	0	Y		3012	4	chr_4:949726-952737 (-)	4413
262146	0	N		968	4	chr_4:949853-950820 (-)	4413's partial sequence
**4414**	0	Y		3012	4	chr_4:953386-956397 (+)	4414
22236	0	Y		3012	4	chr_4:953386-956397 (+)	i.e. 4414
262151	0	N		971	4	chr_4:955303-956273 (+)	4414's partial sequence
23317	2	Y		4215	6	chr_6:1928272-1932765 (-)	i.e. 6575
**6575**	2	Y		4215	6	chr_6:1928356-1932765 (-)	6575
28825	2	Y	N (incorrect ATG)	2610	6	chr_6:1928272-1931188 (-)	6575's partial sequence
29047	1	Y	N (incorrect ATG)	2778	7	chr_7:1859335-1862472 (-)	7305's partial sequence
23631	1	Y		3507	7	chr_7:1859335-1863162 (-)	i.e. 7305
**7305**	1	Y		3507	7	chr_7:1859513-1863162 (-)	7305
263298	0	N		1752	7	chr_7:1859663-1861414 (-)	7305's partial sequence
**7306**	1	Y		3507	7	chr_7:1863894-1867543 (+)	7306
23632	1	Y		3507	7	chr_7:1863894-1867543 (+)	i.e. 7306
263301	0	N		1752	7	chr_7:1865642-1867393 (+)	7306's partial sequence
