# Supplementary material for: Characterization of a Marine Diatom Chitin Synthase Using a Combination of Meta-Omics, Genomics, and Heterologous Expression Approaches
Source: mSystems. 2023 Feb 15;8(2):e01131-22. doi: 10.1128/msystems.01131-22 (PMC10134812; doi:10.1128/msystems.01131-22)
Supplement: TABLE S3 [file msystems.01131-22-s0005.pdf]

Table S3 Amino acid sequences of all the chitin synthases from *Phaeodactylum tricornutum* and *Saccharomyces cerevisiae* .
>Thaps3_J4413

MDETYASGAYDSRGYPVDPSVYSQSQQQQLALHNANQSWGGDDISDLGGRTFESRARSIAGSAALVPKAKYSWASESLGTQSTVMTRR
SLATASVPEPEQTKSKSAAVEGRIWASFAWMVTFPIPNKCIMRPTKDAKQAWREKVALFLIMVSCSVFFVGVFGFVPLLLCKEDTIFAMS
DIWLQTGENWVVVHGTIYDVKDLIYRHPGGVDGIVDFLGKDASKVFPRAPPVTLPQKCLDMEKVESYGLNIEGAENNFTNPTCSSFSELD
VLLGITCHTFAAGSTGVNKFLGDFRRGQLSHTTVGLNGDPEMKWIAIYDRVYDVSTYVDAIRENQEPAVDGEEPSLDHNPAAYLTPTLN
KVIMNSLNADATDLYEALFGSQEYLACLEEMFYVGLLDDNFDSFCYTLNIMMYVMLIFVASLMIIQFLASMIYICPRNRTYTEEDVRSPV
MVMVPCYNEGDNELRKTIKSVLNTTYPDENKVLLMVADGLVTGNGEDMSTPEHLANILGFDIDEFEDDTFEYDCIGVTHTKNRARVYH
GILQKGHKFLKYIVVVKCGLPAEATTSAKPGNRGKRDSQLITIGYFNRIHYGRELTELDSAIQRAMVDLNLQADMVRFLMAIDADTRVDT
MSITHMVYGMDKKEKVLALCGETKVDNKASSWVTMIQVFEYYNSHHLKKAFEAAFGCVTCLPGCFTMYRIIADDGTPLLPGDNVLYEY
SRNDVETLHEKNLYHLGEDRMLTTLLLKHYPDRSLTFIPEAACWTIVPHTFRILLSQRRRWINSTVHNMFELLKVNTMCGVCCVSMKVV
VAIDLIATMILPASYCYAMYLIFLVFFQDLPVSTVLLILYAIIMGVQVVVFILRSRWDYLWWFFIYFTIGLPVFYLILPTYSFWNMDDFSWG
KTRSVGGSAAANAAVADQIEDDEKRGFYEHEKDDNYSYASERSGTSRKSRNSQGSGGSRGDRSNDSRDRSYVSGSRRSMDSRTYRSRD
EYSADSRGSRRSEASESRINF
>Thaps3_J4368

MSRTGRLSDLNSLVNEAKSSNNNGGGGSASASGAASVRSRAPSRASASLGGGPPRFTSAADDASISSFSKSVARVARAAESKDHGGRSV
ASGRRSSASSRGGRSVPNVGGGGGGGGGVVNLNNVPPPRSNQSQASAPRGGNPNNMSSANTYRSSHSSGSGGSRRSTHSDDRTNTTAR
LMSLVQELSQGSNNGSQNFNNNKNNGGGMMNNNMMMQESSRSLQHDNFIHTILSDARSIDEESLSLASGLESRYPMNSNINNSIVPPPP
MRSGGSVSSRNSGPGYTIPHLQMNNNRQDPSVAPSRRSGMSTSSGVASANTSHYSNGQQSYHSATYTATSSTGTGSNEPVGYVMPLGM
GMLPNGTVYSQTSNSMYGGGNNGGDGDGSQCSQDQSMMSGSQASGTMMSGSQTSGGQSTLIGNDHHTMLSGSVPSSAASAPGGSHG
NSYNSNMMMGGGNGDEQDSVMSRSYMSQPLPMNNSQCDTSNMSDASPMLSNHQQVVPRPMYADASFVSGNSRSIANSAALIPKAKF
SWASESLGTQSTVMTRRSMATASVPEPNEQRKSKAVEGRIWASFAWMITFPIPNKCIMRPTKDAKQAWREKVALFVIMVSCSVFFVGVF
GFVPLLLCKEDEIFSMQDIWLQSGENWVVVHGRIYDVKDLIYRHPGGVDGIVDFLGKDASKVFPRSPPIALPRKCLDMEKVDAYSLNVYS
PDNNFTNPTCASFSELDVLLGITCHSFAAGSNGTQKFLGDYERGVLKHTSIGLNEEGIDWISIYDRVYDVSTYVAQIREEQEPAVNGEEPSL
DHNPAAYLTPTLNKVIMNSLGADATDLYEALFGSQEYLLCLEEMFYVGLLDDNFNSFCYTLNIMMYVMLIIVASLMIIQFLASMIYICPRN
RTYTEEDVRSPVMVMVPCYNEGDNELRKTIKSVLNTTYPDENKVLFMVADGLVTGNGEDMSTPEHLANILGFDIDEFEDDTFEYDCIGV
VHTKNRARVYHGILQKGHKFLKYIAIVKCGLPEEATTSAKPGNRGKRDSQLITIGYYNRIHYGRELTELDSAIQRAMVDLNLQADMVRFL
MAIDADTRVDTMSITHMVYGMDKKEKVLALCGETKVDNKASSWVTMIQVFEYYNSHHLKKAFEAAFGCVTCLPGCFTMYRIIADDGTP
LLPGDGVLYEYSRNDVETLHEKNLYHLGEDRMLTTLLLKYYPDRSLTFIPEAACWTIVPHTFRILLSQRRRWINSTVHNMFELLKVNTMC
GVCCVSMKVVVAIDLIATMILPASYCYAMYLLILVFFEDFPVTTVLLILYSIIMGVQVVVFILRSRWEYLWWFLIYFTVGLPVFYLILPTYSF
WNMDDFSWGKTRSVGGSAANNAAAPVEELEQEKDDEKRGLYEHEEDRSFSQARSVKSAHSMRSGVDDDERSTTSSSTSSSGGSDDDE
SSDNDSEYSDESGSDDESEYSQSRASQSQGRY
>Thaps3_J6575

MNHLDSILESIQCSSPRLSGDCSIDTSCDDSGSNNNNSNNDNGSNTVGASSLSSVNNIDTTGNSSAVQSKLSEQQQEWGHRPTASSFSLAT
TAVVANTSPSMNNSAMDEATAAKMYQQAMNDDGDNDVSFPMEAVYEEGSSPARSSRGYNNDVALPMDSLSPSRSNNSNNSPYRQQQ
QQRARTATEESNANLSTTSSTSSTATGGISKHTLVKLMREQITLVRNLTNAQIASKKELEVVKMEKERLEREAREREEGEEHPQQQYNSN
NIKGVGVGGMKMGGIQEEMSQEQSQLRQQYEASKSGGNTPYNNNNNNNNPRHSKLNLPTTIQRGSSDNRSISSRSFYNRFLPTRNNNFN
NSNGPQQSPSYPRRHPDARYYTAHRNRAFSGDTYGEETFGENTLNNGGVSVASTIMPAAIIVGGGVGGMDRMNNNKHSRGPNYTGPQ
AQSNYHNRDKIEITPIPERNVVPPPKSNTCFGTFWWIFSHMCTLFIPDVLLCCIGRNIKVKKKMSKEQKKQLYEMKREAKQAWREKVAIFV
VMLFCSACFIGISGVVPMFLCRETTVFTVDEIQARDRTEDWTVIFGTIYDIKSYISLHPGGDQITSIIGKDASKYFPRRPVGLLPDSCMNPEV
ELTTEAVCEEFDEVDLLMNMHCHSAVGFSGIKRSFGEYERGILAHRASNLKNDPHTDYVMIYNRIYNVTRYIDGITNERTREIEADSENA
YLNEDLTSLIINKRGQDATAVYEALYRDDVALSCLDDLFYIGVLDEPEDILCRVLNYAMYAIMIAIAGVLAVQCLCSLIYLVRMKRTITRD
DTRTKVIVMVPCYNEGDKELRKTIDSVMDTSYPDDNKVLFVVADGNITGKGERKSTPETLSQILGYNINSNDKSYKCKSIGDLTENRAKL
YYGTYKDCGKELKYIVIVKCGVASEKGSPRAGNRGKRDSQLLFTGLLNRFHHGRKLNDLDNGIKNALDHLQMPLDEVRYLMAIDADTRI
DRESISHMTYSMNKNDSVLALCGETKVDNKAQSWVTMIQVFEYYTNHHMKKAFESVFGCVTCLPGCFTMYRLFSDDGRPLLSCDDVY
QRYATNNVKTLHEKNLYHLGEDRMLTTLLLRYFPDMKLSFVPEATCYTIVPHTFSVLLSQRRRWINSTFHNMLELVRVNTMCGVCCFSM
KSIVILDLVATLILPASLIYVGYIVYITFWMGEPLSLLMLVVWGIVVGVQVVVFLLRSRWDYWWWFLVFILLGVPVFYFILPIYSFWHMDD
FSWGATRQVSGQAAVTSSKSKDDTICSDRNSPMGFEVTADEHAKTAHRNAAAAQKQRAPSAPLRSKPPRHTSSLYQTSTPIDVDEEGGR
YSASHNAAHRYGESNPVDLDAVSVDNFTLEASVNTKTTFDPEKYSDEEEAKRIRRLRRAAC
>Thaps3_J7305

MSSRHDDSSMSSFSAGLSAYDAAEPKPMNSAANSVRSSKSKSSRSNYSPAPPPPRPAGSSVANSYRSQPKSVDGRSSQSVASGLSLGSAG
RLSRQVPMDDDDQSNNTARLVGLVNELTRNGGEDDEYEMERRRQMEEERYRLQDEIRHQMDDDNYSDHHSRQLSRRSRDPESDEASR
RSNMSGVPPPPRSVRSGHSQSHQSRSRYDPDGMSHDFGADDPDGRHYAPGLNNHGGVPSVHTGIPPMDEESHQGSQSYYGEDQSTLRS
MKEEESYYSRDSRSRQSSTVRSVPAPPPGLSAEMSQFSGAGEDESYYSRQSRGSYQSSKKARPVPVKASNENKRAMWWYNFSRCVTFPI
PDTCIRKDDPEAKQAWREKVAICFCVFLVSGFFVGIFGFVPVLLCRERTVYTLQNVQERTNEEWIVLNGYVYDVGGLFNKHPGGPAGIEA
FLNDDASRMFPRLPPALLPPMCLNKAKIEESEELQSTDKVCAQMTDEDKKNGVPCHTFVTGINETAKYMGEFEKGILAHDGGVLLESPFT
FWVSIHDRVYNVTDYVNNIRNDQTKQIEKDHPMAYLEPTLNNLVINKLNEDATELFLSVYPDDRILMCMDELFYVGIIDTRFDVVCFVLN
ILMYFLLGFVALIMVAQMLCSLMYIAKTTRTYTEEDTQDQVIVMVPCYNEGGQELIKTIDSVLGTTYPEENKVVFIIADGVVTGAGQELST
PEHLAEILGFEMDPENDELYEYDSIGLLTTNRARVYHGYYEVDDKSLKYLVVVKAGLPMEQAGSAKPGNRGKRDSQLVIMGYFNRIYH
GRELNELDAAIEDALIDLNMQSDDMRLLMTIDADTRVDELSITHMTYAMNQNDRILALCGETKVDNKWQSWVTMMQVFEYYNNHHM
KKAFESAFGCVTCLPGCFTMYRILNDDGKPLLADDHVYAEYLRNDIDSLHEQNLFHLGEDRMLTTLLLHFFPGMFLSYVPEAQCFTIVPHT
LRILMSQRRRWINSTYHNLLELTKVKTMCGVCCCSMKTVVYLDLVACMILPASTVYAAYLVFLVAAGKTQFSLLLLVLYGLLIGVQLVVF
IVRSRYDFFFWFIYFTLVGVPVFYFLLPLYAFWHMDDFSWGETRKVAAMKDSFKNPQAMQAQAAATDDEDESDDESEAYSRRSSGSYS
RDSRSKRSMS
>ScCHS1

MSDQNNRSRNEYHSNRENEPSYELQNAHSGLFHSSNEELTNRNQRYTNQNASMGSFTPVQSLQFPEQSQQTNMLYNGDDGNNNTIND
NERDIYGGFVNHYRQRPPPATAEYNDVFNTNSQQLPSEHQYNNVPSYPLPSINVIQTTPELIHNGSQTMATPIERPFFNENDYYYNNRNS
RTSPSIASSSDGYADQEARPILEQPNNNMNSGNIPQYHDQPFGYNNGYHGLQAKDYYDDPEGGYIDQRGDDYQINSYLGRNGEMVDPY
DYENSLRHMTPMERREYLHDDNRPVNDGKEELDSLKSGYSHRDLGEYDKDDFSRDDEYDDLNTIDKLQFQANGVPASSSVSSIGSKESD
IIVSNDNLTANRALKRSGTEIRKFKLWNGNFVFDSPISKTLLDQYATTTENANTLPNEFKFMRYQAVTCEPNQLAEKNFTVRQLKYLTPRE
TELMLVVTMYNEDHILLGRTLKGIMDNVKYMVKKKNSSTWGPDAWKKIVVCIISDGRSKINERSLALLSSLGCYQDGFAKDEINEKKVA
MHVYEHTTMINITNISESEVSLECNQGTVPIQLLFCLKEQNQKKINSHRWAFEGFAELLRPNIVTLLDAGTMPGKDSIYQLWREFRNPNVG
GACGEIRTDLGKRFVKLLNPLVASQNFEYKMSNILDKTTESNFGFITVLPGAFSAYRFEAVRGQPLQKYFYGEIMENEGFHFFSSNMYLAE
DRILCFEVVTKKNCNWILKYCRSSYASTDVPERVPEFILQRRRWLNGSFFASVYSFCHFYRVWSSGHNIGRKLLLTVEFFYLFFNTLISWFS
LSSFFLVFRILTVSIALAYHSAFNVLSVIFLWLYGICTLSTFILSLGNKPKSTEKFYVLTCVIFAVMMIYMIFCSIFMSVKSFQNILKNDTISFEG
LITTEAFRDIVISLGSTYCLYLISSIIYLQPWHMLTSFIQYILLSPSYINVLNIYAFCNVHDLSWGTKGAMANPLGKINTTEDGTFKMEVLVSS
SEIQANYDKYLKVLNDFDPKSESRPTEPSYDEKKTGYYANVRSLVIIFWVITNFIIVAVVLETGGIADYIAMKSISTDDTLETAKKAEIPLMT
SKASIYFNVILWLVALSALIRFIGCSIYMIVRFFKKVTFR
>ScCHS2

MTRNPFMVEPSNGSPNRRGASNLSKFYANANSNSRWANPSEESLEDSYDQSNVFQGLPASPSRAALRYSPDRRHRTQFYRDSAHNSPV
APNRYAANLQESPKRAGEAVIHLSEGSNLYPRDNADLPVDPYHLSPQQQPSNNLFGSGRLYSQSSKYTMSTTSTTAPSLAEADDEKEKYL
TSTTSYDDQSTIFSADTFNETKFELNHPTRQQYVRRANSESKRRMVSDLPPPSKKKALLKLDNPIPKGLLDTLPRRNSPEFTEMRYTACTV
EPDDFLREGYTLRFAEMNRECQIAICITMYNEDKYSLARTIHSIMKNVAHLCKREKSHVWGPNGWKKVSVILISDGRAKVNQGSLDYLA
ALGVYQEDMAKASVNGDPVKAHIFELTTQVSINADLDYVSKDIVPVQLVFCLKEENKKKINSHRWLFNAFCPVLQPTVVTLVDVGTRLN
NTAIYRLWKVFDMDSNVAGAAGQIKTMKGKWGLKLFNPLVASQNFEYKISNILDKPLESVFGYISVLPGALSAYRYRALKNHEDGTGPL
RSYFLGETQEGRDHDVFTANMYLAEDRILCWELVAKRDAKWVLKYVKEATGETDVPEDVSEFISQRRRWLNGAMFAAIYAQLHFYQIW
KTKHSVVRKFFLHVEFLYQFIQMLFSWFSIANFVLTFYYLAGSMNLVIKHGEALFIFFKYLIFCDLASLFIISMGNRPQGAKHLFITSMVILSIC
ATYSLICGFVFAFKSLASGTESHKIFVDIVISLLSTYGLYFFSSLMYLDPWHMFTSSIQYFLTLPAFTCTLQIFAFCNTHDVSWGTKGSTQES
KQLSKAIVVQGPDGKQIVETDWPQEVDKKFLEIKSRLKEPEFEESSGNEKQSKNDYYRDIRTRIVMIWMLSNLILIMSIIQVFTPQDTDNGY
LIFILWSVAALAAFRVVGSMAFLFMKYLRIIVSYRNKVEGSGSWEVSKLDLPNVFHKKG
>ScCHS3

MTGLNGDDPDDYYLNLNQDEESLLRSRHSVGSGAPHRQGSLVRPERSRLNNPDNPHFYYAQKTQEQMNHLDVLPSSTGVNPNATRRSG
SLRSKGSVRSKFSGRETDSYLLQDMNTTDKKASVKISDEGVAEDEFDKDGDVDNFEESSTQPINKSIKPLRKETNDTLSFWQMYCYFITF
WAPAPILAFCGMPKKERQMAWREKVALISVILYIGAIVAFLTFGFTKTVCSSSKLRLKNNEVSTEFVVINGKAYELDTSSRSGIQDVEVDS
DTLYGPWSDAGKDASFLFQNVNGNCHNLITPKSNSSIPHDDDNNLAWYFPCKLKNQDGSSKPNFTVENYAGWNCHTSKEDRDAFYGLK
SKADVYFTWDGIKNSSRNLIVYNGDVLDLDLLDWLEKDDVDYPVVFDDLKTSNLQGYDLSLVLSNGHERKIARCLSEIIKVGEVDSKTV
GCIASDVVLYVSLVFILSVVIIKFIIACYFRWTVARKQGAYIVDNKTMDKHTNDIEDWSNNIQTKAPLKEVDPHLRPKKYSKKSLGHKRAS
TFDLLKKHSSKMFQFNESVIDLDTSMSSSLQSSGSYRGMTTMTTQNAWKLSNENKAVHSRNPSTLLPTSSMFWNKATSSPVPGSSLIQSL
DSTIIHPDIVQQPPLDFMPYGFPLIHTICFVTCYSEDEEGLRTTLDSLSTTDYPNSHKLLMVVCDGLIKGSGNDKTTPEIALGMMDDFVTPP
DEVKPYSYVAVASGSKRHNMAKIYAGFYKYDDSTIPPENQQRVPIITIVKCGTPAEQGAAKPGNRGKRDSQIILMSFLEKITFDERMTQLE
FQLLKNIWQITGLMADFYETVLMVDADTKVFPDALTHMVAEMVKDPLIMGLCGETKIANKAQSWVTAIQVFEYYISHHQAKAFESVFGS
VTCLPGCFSMYRIKSPKGSDGYWVPVLANPDIVERYSDNVTNTLHKKNLLLLGEDRFLSSLMLKTFPKRKQVFVPKAACKTIAPDKFKVL
LSQRRRWINSTVHNLFELVLIRDLCGTFCFSMQFVIGIELIGTMVLPLAICFTIYVIIFAIVSKPTPVITLVLLAIILGLPGLIVVITATRWSYLW
WMCVYICALPIWNFVLPSYAYWKFDDFSWGDTRTIAGGNKKAQDENEGEFDHSKIKMRTWREFEREDILNRKEESDSFVA
