# Supplementary material for: Characterization of a Marine Diatom Chitin Synthase Using a Combination of Meta-Omics, Genomics, and Heterologous Expression Approaches
Source: mSystems. 2023 Feb 15;8(2):e01131-22. doi: 10.1128/msystems.01131-22 (PMC10134812; doi:10.1128/msystems.01131-22)
Supplement: TABLE S4 [file msystems.01131-22-s0006.pdf]

Table S4 Sequence analysis of chitin synthase 1 from *Thalassiosira pseudonana.*

**Name**	**TpCHS1**
JGI ID	4413
Length of genomic sequence	3,012 bp
Chromosomal location	4
Number of exons	1
Length of CDS	3,012 bp
GC content	50%
Size of protein	1,003 AA
Molecular weight	113.1 kDa
Isoelectric point	5.37
Alpha helix	34.80%
Random coil	35%
Extended strand	20.90%
Beta turn	9.30%
Transmembrane helices	5
SignalP	0
GPI anchor	0
High protein amount	Cytokinesis/Cell wall
