# Supplementary material for: Characterization of a Marine Diatom Chitin Synthase Using a Combination of Meta-Omics, Genomics, and Heterologous Expression Approaches
Source: mSystems. 2023 Feb 15;8(2):e01131-22. doi: 10.1128/msystems.01131-22 (PMC10134812; doi:10.1128/msystems.01131-22)
Supplement: TABLE S5 [file msystems.01131-22-s0007.pdf]

Table S5 Diatom TpCHS1 homologous sequences retrieved in MMETSP, PhycoCosm Bacillariophyta, and PLAZA diatoms 1.0 databases.

Data source: MMETSP

Class	Order (Homologous sequence No.)	Species (Homologous sequence No.)
Coscinodiscophyceae	**Thalassiosirales (420)**	*Thalassiosira rotula* (34)
		Thalassiosira oceanica (39)
		Thalassiosira weissflogii (146)
		Thalassiosira punctigera (5)
		Thalassiosira gravida (12)
		Thalassiosira antarctica (19)
		Thalassiosira miniscula (18)
		Thalassiosira sp. (15)
		Skeletonema marinoi (75)
		Skeletonema costatum (17)
		Skeletonema dohrnii (13)
		Skeletonema menzelii (9)
		Skeletonema grethea (5)
		Skeletonema japonicum (5)
		Cyclotella meneghiniana (4)
		Detonula confervacea (4)
Mediophyceae	**Eupodiscales (22)**	*Odontella sinensis* (18)
		Odontella aurita (4)
	**Lithodesmiales (13)**	*Ditylum brightwellii* (9)
		Helicotheca tamensis (2)
		Lithodesmium (2)
	**Cymatosirales (10)**	*Extubocellulus spinifer* (4)
		Minutocellus polymorphus (6)
	**Triceratiales (4)**	*Triceratium dubium* (4)
Fragilariophyceae	**Thalassionemales (5)**	*Thalassiothrix antarctica* (2)
		Thalassionema frauenfeldii (1)
		Thalassionema nitzschioides (2)
	**Fragilariales (1)**	*Synedropsis recta* (1)
Bacillariophyceae	**Naviculales (12)**	*Fistulifera solaris* (8)
		Craspedostauros australis (2)
		Stauroneis constricta (2)

Data source: PhycoCosm

Class	Order (Homologous sequence No.)	Species (Homologous sequence No.)
Coscinodiscophyceae	**Thalassiosirales (320)**	*Thalassiosira pseudonana* (70)
		Cyclotella cryptica (8)
		Minidiscus variabilis (242)
Bacillariophyceae	**Naviculales (14)**	*Fistulifera solaris* (8)
		Phaeodactylum tricornutum (6)
	**Bacillariales (6)**	*Nitzschia hildebrandi* (6)

Data source: PLAZA

Class	Order (Homologous sequence No.)	Species (Homologous sequence No.)
Coscinodiscophyceae	**Thalassiosirales (23)**	*Thalassiosira pseudonana* (8)
		Cyclotella cryptica (8)
		Thalassiosira oceanica (7)
Bacillariophyceae	**Naviculales (8)**	*Fistulifera solaris* (8)
	**Fragilariales (1)**	*Synedra acus* (1)

Note: The species taxonomy was referred to https://www.uniprot.org/taxonomy/
