# Supplementary figures and images for: Characterization of a Marine Diatom Chitin Synthase Using a Combination of Meta-Omics, Genomics, and Heterologous Expression Approaches
Source: mSystems. 2023 Feb 15;8(2):e01131-22. doi: 10.1128/msystems.01131-22 (PMC10134812; doi:10.1128/msystems.01131-22)

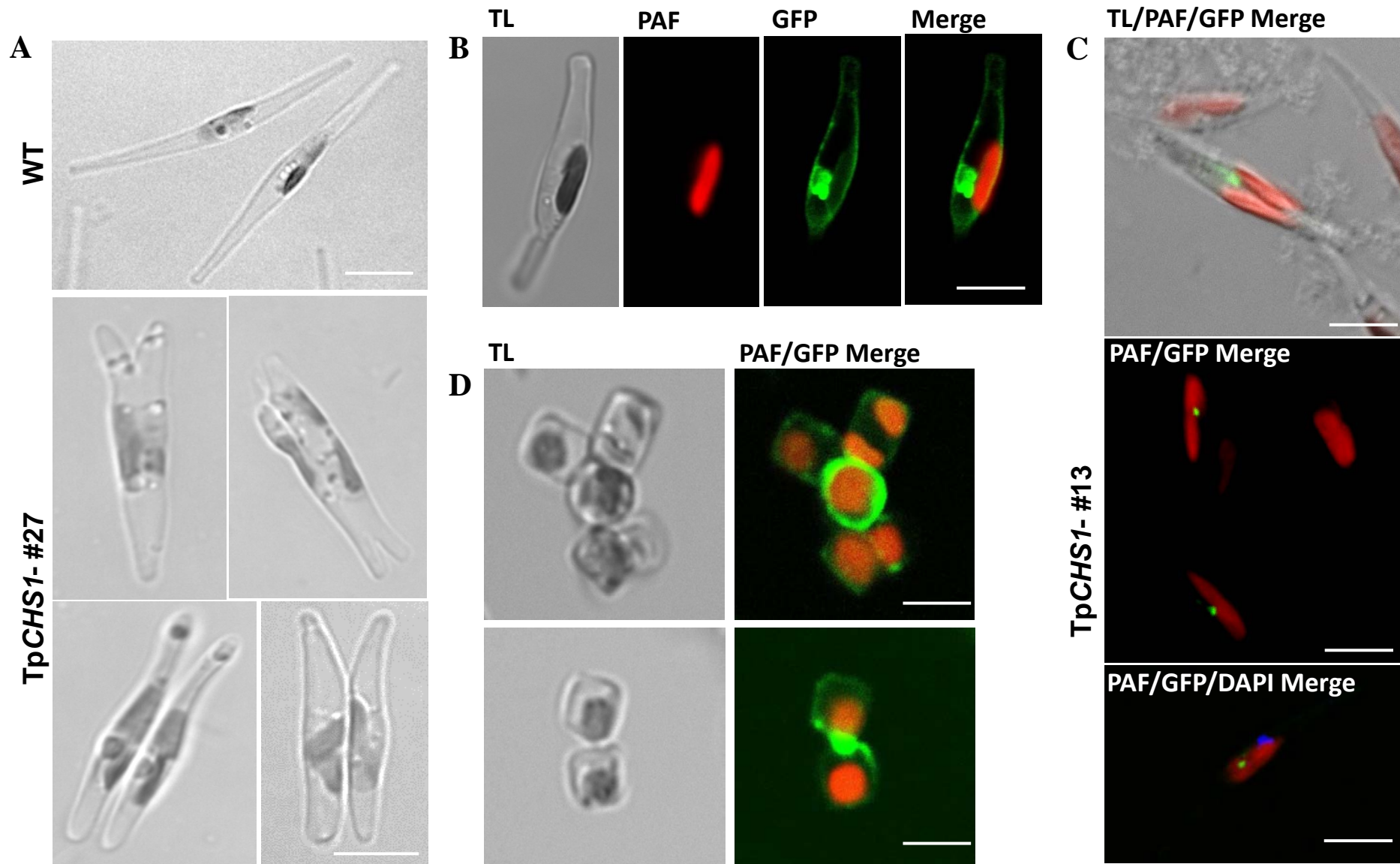

Supplemental Figure 2

Supplement: FIG S2 [file msystems.01131-22-s0002.pdf]
