# Supplementary material for: Characterization of a Marine Diatom Chitin Synthase Using a Combination of Meta-Omics, Genomics, and Heterologous Expression Approaches
Source: mSystems. 2023 Feb 15;8(2):e01131-22. doi: 10.1128/msystems.01131-22 (PMC10134812; doi:10.1128/msystems.01131-22)
Supplement: TABLE S7 [file msystems.01131-22-s0009.pdf]

Table S7 Detailed protocols for Tp*CHS1-pDDGFP-2* and Tp*CHS1-pPhaT1-eGFP* plasmids construction.

**For pDDGFP-2**
Tp*CHS1* gene synthesis was conducted by GeneArt™ with codons optimized for yeast (Thermo Fisher, USA). Homologous recombination was used to construct Tp*CHS1-pDDGFP-2* fusion plasmid. One pair of primers was designed: Tp*CHS1*-HR-Fw: accccggattctagaactagtggatcccccATGGACGAAACCTACGCTTC; Tp*CHS1*-HR-Rv: aaattgaccttgaaaatataaattttccccGAAGTTGATTCTAGATTCAG. The PCR protocol for amplifying Tp*CHS1* gene with overhangs was as follows: 94 °C, 5 min; 35 cycles of 94 °C, 30 s, 58 °C, 30 s, 72 °C 2 min; 72 °C 10 min. pDDGFP-2 vector with URA selection marker was digested with *Sma*I, producing blunt ends. The amplified Tp*CHS1* products (5 µL of 150 ng/µL) and linearized pDDGFP-2 vector (3 µL of 25 ng/µL) were then transformed into 50 µL *of S. cerevisiae* BY4742 (*chs1Δ::KanMX4*, *chs3Δ::HIS3* and *pep4Δ::LYS2*) competent cells, with inoculation for 30 min at 30 °C, prior to heat shock at 42 °C for 25 min. More details could be referred to BOX 1 in the paper by Drew et al. (2008).
**For pPhaT1-eGFP**
Gibson Assembly strategy was applied to construct expression plasmid (NEB, USA) (Gibson *et al.*, 2010). Tp*CHS1* full-length sequence was amplified from the *T. pseudonana* cDNA products with the following primers: Tp*CHS1*-fcpA-Fw, 5'-caaatttgtctgccgtttcgagaaATGGACGAAACCTACGCCAG-3'; Tp*CHS1*-eGFP-Rv, 5'-cctcgcccttgctcaccatAAAGTTGATTCTAGACTCGG-3'. Overlapping PCR fragments were amplified for pPhaT1-eGFP vector with three pairs of primers: eGFP-Fw: 5'-ATGGTGAGCAAGGGCGAGG-3', sh ble-Rv:5'- CTGCTCCTCGGCCACGAAG-3'; sh ble-Fw:5'- CTTCGTGGCCGAGGAGCAG-3', Amp-Rv: 5'-GATAAATCTGGAGCCGGTGAGCGTGGG-3'; Amp-Fw: 5'-CCCACGCTCACCGGCTCCAGATTTATC-3', fcpA-Rv: 5'-TTCTCGAAACGGCAGACAAATTTG-3'. High-fidelity PCR products were amplified using Phusion DNA polymerase (Thermo, USA). The Gibson reaction was set up with 50 ng each for three linearized pPhaT1-eGFP vector fragments and 150-250 ng for Tp*CHS1* product. The mixture with 4 fragments were then placed in a thermal cycler for 4 h at 50 °C.
