# Supplementary material for: Characterization of a Marine Diatom Chitin Synthase Using a Combination of Meta-Omics, Genomics, and Heterologous Expression Approaches
Source: mSystems. 2023 Feb 15;8(2):e01131-22. doi: 10.1128/msystems.01131-22 (PMC10134812; doi:10.1128/msystems.01131-22)
Supplement: TABLE S8 [file msystems.01131-22-s0010.pdf]

Table S8 List of qRT-PCR primers for Tp*CHS1* gene, cell cycle marker genes and housekeeping gene.

**Gene**	**Protein ID**	**Forward primer**	**Reverse primer**	**Full name**
CHS1	Thaps3_J4413	ACTTGCTCTACACAACGCCA	GTCACCATCCAAGCGAAGGA	Chitin synthase
CYCB1	Phatr3_J46095	TCCTGGTCCGCTACTTGAAAG	GCTGGCTGGGAAGATAACGC	B-type cyclin
CYCP6	Phatr3_J6231	AGGTGCTTGCTGCTGTTC	ACGAGGCATACTTGTGAATCC	P-type cyclin
E2F1	Phatr3_J43065	CCCTAAGCGGCGGATTTACG	AAGCGACGAGCCAAGAAGAAGC	Transcription factor E2F1
RPS	Phatr3_J10847	CGAAGTCAACCAGGAAACCAA	GTGCAAGAGACCGGACATACC	Ribosomal protein small subunit 30S
