# Supplementary material for: Characterization of a Marine Diatom Chitin Synthase Using a Combination of Meta-Omics, Genomics, and Heterologous Expression Approaches
Source: mSystems. 2023 Feb 15;8(2):e01131-22. doi: 10.1128/msystems.01131-22 (PMC10134812; doi:10.1128/msystems.01131-22)
Supplement: TABLE S6 [file msystems.01131-22-s0008.pdf]

Table S6 All 36 sequences for the phylogenetic tree construction searched in MMETSP, PhycoCosm and PLAZA databases with high sequence similarity to TpCHS1.

>Thaps3_J4413/TpCHS1

[illegible]

>Thaps3_J4368

[illegible]

>Thaps3_J6575

[illegible]

>Thaps3_J7305

[illegible]

>D2_CAMPEP_0201665904 / Thalassiosira gravida, Strain GMp14c1 /

[illegible]

>D2_CAMPEP_0196133396 / Thalassiosira rotula, Strain GSO102 /

[illegible]

>D2_CAMPEP_0203536538 / Thalassiosira weissflogii, Strain CCMP1010 /

[illegible]

>D2_CAMPEP_0201866970 / Thalassiosira antarctica, Strain CCMP982 /

[illegible]

>D2_CAMPEP_0172298518 / Detonula confervacea, Strain CCMP 353 /

[illegible]

>D2_CAMPEP_0183744108 / Thalassiosira miniscula, Strain CCMP1093 /

[illegible]

>D2_CAMPEP_0206711446 / Thalassiosira oceanica, Strain CCMP1005 /

[illegible]

>D2_CAMPEP_0172531652 / Thalassiosira punctigera, Strain Tpunct2005C2 /

[illegible]

>D2_CAMPEP_0181106326 / Thalassiosira sp., Strain NH16 /

[illegible]

>D2_CAMPEP_0115905852 / Skeletonema marinoi, Strain SM1012Hels-07 /

[illegible]

>D2_CAMPEP_0201738616 / Skeletonema japonicum, Strain CCMP2506 /

[illegible]

>D2_CAMPEP_0113373600 / Skeletonema costatum, Strain 1716 /

[illegible]

>D2_CAMPEP_0183648348 / Skeletonema menzelii, Strain CCMP793 /

[illegible]

>D2_CAMPEP_0201711188 / Skeletonema grethea, Strain CCMP 1804 /

[illegible]

>D2_CAMPEP_0195242090 / Skeletonema dohrnii, Strain SkelB /

[illegible]

>D2_CAMPEP_0172292322 / Cyclotella meneghiniana, Strain CCMP 338 /

[illegible]

>CAMPEP_0180929722 / Ditylum brightwellii, Strain Pop1 (SS4) /

[illegible]

>D1_CAMPEP_0181074656 / Minutocellus polymorphus, Strain NH13 /

[illegible]

>D3_CAMPEP_0197432758 / Triceratium dubium, Strain CCMP147 /

[illegible]

>CAMPEP_0183303582 / Odontella sinensis, Strain Grunow 1884 /

[illegible]

>CAMPEP_0113538006 / Odontella /

[illegible]

>Pen_Raph_CAMPEP_0119571204 / Stauroneis constricta, Strain CCMP1120 /

[illegible]

>Pen_Ara_CAMPEP_0119017256 / Synedropsis recta cf, Strain CCMP1620 /

[illegible]

>Pen_Ara_CAMPEP_0194181470 / Thalassiothrix antarctica, Strain L6-D1 /

[illegible]

>Pen_Ara_CAMPEP_0194246588 / Thalassionema nitzschioides, Strain L26-B /

[illegible]

>Pen_Raph_CAMPEP_0198134994 / Craspedostauros australis, Strain CCMP3328 /

[illegible]

>Pen_Ara_CAMPEP_0178917392 / Thalassionema frauenfeldii, Strain CCMP 1798 /

[illegible]

>CAMPEP_0185741788 / Helicotheca tamensis, Strain CCMP826 /

[illegible]

>Cyccr1/8218

[illegible]

>Mintr2/745389

[illegible]

>Nithil2/4306

[illegible]

>sva00G260910

[illegible]
